# Transpulmonary thermodilution using femoral indicator injection: a prospective trial in patients with a femoral and a jugular central venous catheter

**DOI:** 10.1186/cc9030

**Published:** 2010-05-25

**Authors:** Bernd Saugel, Andreas Umgelter, Tibor Schuster, Veit Phillip, Roland M Schmid, Wolfgang Huber

**Affiliations:** 1 II. Medizinische Klinik und Poliklinik, Klinikum rechts der Isar der Technischen Universität München, Ismaninger Str. 22, 81675 München, Germany; 2 Institut für Medizinische Statistik und Epidemiologie. Lehrstuhl für Medizinische Informatik, Klinikum rechts der Isar der Technischen Universität München, Ismaninger Str. 22, 81675 München, Germany

## Abstract

**Introduction:**

Advanced hemodynamic monitoring using transpulmonary thermodilution (TPTD) is established for measurement of cardiac index (CI), global end-diastolic volume index (GEDVI) and extra-vascular lung water index (EVLWI). TPTD requires indicator injection via a central venous catheter (usually placed via the jugular or subclavian vein). However, superior vena cava access is often not feasible due to the clinical situation. This study investigates the conformity of TPTD using femoral access.

**Methods:**

This prospective study involved an 18-month trial at a medical intensive care unit at a university hospital. Twenty-four patients with both a superior and an inferior vena cava catheter at the same time were enrolled in the study.

**Results:**

TPTD-variables were calculated from TPTD curves after injection of the indicator bolus via jugular access (TPTDjug) and femoral access (TPTDfem). GEDVIfem and GEDVIjug were significantly correlated (r_m _= 0.88; *P *< 0.001), but significantly different (1,034 ± 275 vs. 793 ± 180 mL/m^2^; *P *< 0.001). Bland-Altman analysis demonstrated a bias of +241 mL/m^2 ^(limits of agreement: -9 and +491 mL/m^2^). GEDVIfem, CIfem and ideal body weight were independently associated with the bias (GEDVIfem-GEDVIjug). A correction formula of GEDVIjug after femoral TPTD, was calculated. EVLWIfem and EVLWIjug were significantly correlated (r_m _= 0.93; *P *< 0.001). Bland-Altman analysis revealed a bias of +0.83 mL/kg (limits of agreement: -2.61 and +4.28 mL/kg). Furthermore, CIfem and CIjug were significantly correlated (r_m _= 0.95; *P *< 0.001). Bland-Altman analysis demonstrated a bias of +0.29 L/min/m^2 ^(limits of agreement -0.40 and +0.97 L/min/m^2^; percentage-error 16%).

**Conclusions:**

TPTD after femoral injection of the thermo-bolus provides precise data on GEDVI with a high correlation, but a self-evident significant bias related to the augmented TPTD-volume. After correction of GEDVIfem using a correction formula, GEDVIfem shows high predictive capabilities for GEDVIjug. Regarding CI and EVLWI, accurate TPTD-data is obtained using femoral access.

## Introduction

Advanced hemodynamic monitoring is a cornerstone of intensive care. Transpulmonary thermodilution (TPTD) is established for the measurement of cardiac index (CI), preload, volume responsiveness and pulmonary hydration in critically ill intensive care unit (ICU) patients [1-9]. For the assessment of volume responsiveness TPTD provides *volumetric *parameters such as global end-diastolic volume index (GEDVI) that can be used regardless of sinus rhythm and controlled ventilation [2,4-6].

In addition, TPTD accurately allows measurement of extra-vascular lung water index (EVLWI) to quantify the degree of pulmonary edema [8,10-21]. TPTD is based on the injection of a cold saline bolus through a central venous catheter (CVC) in the central venous circulation. The subsequent change in blood temperature is picked up by a thermistor located in the tip of a catheter usually placed in the descending aorta through the femoral artery. A thermodilution curve is created and the hemodynamic parameters are obtained after its analysis. CI, GEDVI and EVLWI are calculated using three main values determined by contour analysis of the thermodilution curve: area under the curve, mean transit time, and down-slope time. Mean transit time describes the time until half of the injected saline bolus has passed the thermistor. Down-slope time describes the duration of the exponential decrease of the dilution curve and allows calculation of the largest of several series-connected chambers and finally of EVLWI.

Usually the CVC for TPTD is placed via the jugular or subclavian vein. Superior vena cava access was a prerequisite in the validation studies for TPTD. However, superior vena cava access is often not feasible due to the clinical situation. Clinical circumstances such as thrombosis of the jugular vein, polytrauma, burns, use of the superior vena cava access for Shaldon catheters and infection of previous puncture sites might necessitate femoral access. In these situations the CVC has to be inserted in the inferior vena cava via the femoral vein. Moreover, femoral venous catheterization provides a rapid way in emergency situations to obtain central venous vascular access. A review of the literature clearly demonstrates that the use of femoral vein access for central venous access is often necessary. In recent studies investigating the influence of the insertion site on CVC colonisation and bloodstream infections femoral access was used in about 20 to 35% of all catheter insertions [22,23].

To the best of our knowledge, only one report on 11 patients with different numbers of measurements per patient investigated the accuracy of TPTD variables derived after central venous injection via the femoral access [24].

Therefore, it was the aim of our study to prospectively investigate the conformity of femoral versus jugular access TPTD in 24 critically ill patients with an identical number of two pairs of TPTD measurements in each patient.

## Materials and methods

### Patients

Between January 2008 and June 2009, 24 patients treated in the medical ICU of a German university hospital (Klinikum rechts der Isar der Technischen Universität München, Munich, Germany) were included in the study. All patients had both a superior and an inferior vena cava catheter at the same time for clinical reasons unrelated to the study. A total of 96 TPTD measurements were analyzed (48 TPTDs via femoral access compared to 48 TPTDs via jugular access; four TPTDs per patient, two TPTDs per patient via femoral venous access and two TPTDs per patient via jugular venous access). Each TPTD measurement represents the mean of three consecutive TPTD indicator injections. Between June 2009 and October 2009, five more patients were separately studied to evaluate the correction formula for GEDVI derived from the first 24 patients in a different study population. These five patients were not included in the primary study analysis but served as a control group. In these five patients a total of 20 TPTD measurements were analyzed (10 TPTDs via femoral access compared to 10 TPTDs via jugular access; four TPTDs per patient, two TPTDs per patient via femoral venous access and two TPTDs per patient via jugular venous access). The study was approved by the local ethics committee (Technical University of Munich, project number 2074/08). Informed consent was obtained according to the Declaration of Helsinki.

### TPTD measurements

TPTD was performed using a 5-French thermistor-tipped arterial line (Pulsiocath, Pulsion Medical Systems AG, Munich, Germany) that was inserted in the abdominal aorta through the femoral artery and connected to a hemodynamic monitor (PiCCO-Plus, software version 7.1; PiCCO-2, software version 1.3.0.8; Pulsion Medical Systems AG). Using the superior vena cava catheter and the inferior vena cava catheter, respectively, central venous pressure (CVP) was recorded throughout the respiratory cycle and measured at end-expiration. In all patients the same type of 4-lumen CVC was used for femoral and jugular access (MultiCath 4 Expert, 8.5 French; Vygon GmbH & Co. KG, Aachen, Germany). After insertion of the catheter the correct tip position of the jugular CVC was verified by x-ray. Femoral CVCs were completely inserted. According to the manufacturer's recommendation, via the jugular and femoral access, respectively, 15 mL cold saline 0.9% were injected through the distal lumen of the catheter (priming lumen of the distal catheter lumen: 0.38 mL). Based on TPTD, CI, GEDVI and EVLWI were determined [8,9,20,25-27]. Each PiCCO measurement represents the mean of three consecutive thermodilution measurements. Measurement procedures were performed twice for each patient with a mean time interval of 9.54 ± 7.27 hours (minimum one hour, maximum 24 hours). One measurement procedure consisted of three injections via jugular vein and three injections via femoral vein within a maximum of 15 minutes. During the measurement procedures no changes were made in catecholamine therapy or intravascular volume administration, respirator settings and the patients' position. The CVC site for the initial injection (jugular or femoral vein) was selected randomly. Hemodynamic parameters, determined using TPTD via superior vena cava access, were compared with those derived from TPTD via inferior vena cava access. Global end-diastolic volume (GEDV) was indexed for body surface area and extra-vascular lung water (EVLW) was indexed for predicted body weight.

### Statistical analysis

Bivariate correlation of quantitative data (means of paired measurements per patient) was assessed using Spearman correlation coefficient (r_m_).

Normality of data was assessed both, descriptively (by investigating histograms and QQ-plots) and by using statistical tests (Shaphiro-Wilk test). There were no considerable violations of normality. Since Spearman rank correlation describes the monotonicity of bivariate relationship and is not sensitive to high leverage points this measure was preferred to the ordinary linear correlation coefficient.

With a total number of 24 patients modest bivariate correlations of about |r| (absolute amount of r) = 0.50 or higher would have been detectable with 80% power at a two sided level of significance of 5%.

The percentage errors of hemodynamic parameters were calculated as demonstrated by Critchley [28].

The root mean square coefficient of variation (RMSCV) was determined to assess variability of repeated single TPTD measurements. Since RMSCV is independent of the level of measurement it provides an appropriate quantity for a comparative evaluation of measurement stability.

To illustrate differences of TPTD parameters derived after femoral and jugular injection in dependence of mean measurement levels Bland-Altman-plots were provided. In this term, agreement between two measurement methods was evaluated by calculating the systematically error (bias) with the 95% limits of individual agreement as bias ± 2 standard deviation (SD). Random effects models were used to estimate the within-subject variation and to achieve estimates of total variability for Bland-Altman analysis considering the issue of repeated measures per subject [29].

By the use of multiple linear regression analysis, prediction models for jugular TPTD parameters were developed. For this purpose, potentially predictive capability of femoral parameters was assessed by a general estimation equation (GEE) model [30]. The GEE approach properly reflects the structure of repeated data and takes correlation of repeated (two pairs of) measurements per patient into account. No consideration of repeated data issue would yield to overly optimistic estimates (smaller standard errors) and therefore potentially to inappropriate conclusions.

Parameters which showed a substantial linear correlation (indicated by a *P*-value for the regression coefficient <0.10 and leading to an elevated adjusted r^2^, respectively) within the multivariable GEE model, were considered in the final prediction model based on means of paired measurements per patient.

Means were reported with standard deviations (mean ± SD) and regression coefficients (slopes) from linear GEE models were depicted with standard errors (b ± SE). Statistical analysis was performed using software (SPSS. version 16; SPSS inc., Chicago, IL, USA).

## Results

### Patients and patients' characteristics

A total of 96 TPTDs (48 via femoral access, 48 via jugular access) of 24 critically ill ICU patients were enrolled in this study. Basic demographic data and reasons for ICU admission are shown in Table 1.

**Table 1:** Patients' characteristics, cardiopulmonary characteristics, reason for intensive care unit admission and vascular access

	Mean (SD	Range
Patients' characteristics		
Sex, n	15 men, 9 women	
Age, years	67.4 (9.5	46 to 88
Height, cm	171 (8	156 to 187
Weight, kg	75.3 (18.5	45.0 to 110.0
Body surface area, m^2^	1.87 (0.27	1.40 to 2.40
Body mass index, kg/m^2^	25.5 (4.8	16.5 to 35.9
Ideal body weight, kg	62.8 (8.5	47.6 to 78.3
Normal body weight, kg	71.0 (8.1	56.0 to 87.0
Predicted body weight, kg	65.6 (9.3	48.8 to 81,5
Adjusted body weight, kg	69.1 (11.9	49.3 to 91.7
SAPS II	41.3 (10.9	26 to 66
TISS	20.8 (6.1	9 to 34
ICU survival, n	11 yes, 13 no	

**Cardiopulmonary characteristics**		

Heart rate, beats per minute	93.8 (16.7	61 to 125
Mean arterial pressure, mmHg	85.5 (15.0	60 to 122
CIjug-avg, L/min/m^2^	4.03 (1.13	2.30 to 7.30
CIfem-avg, L/min/m^2^	4.31 (1.18	2.41 to 7.45
GEDVIjug-avg, mL/m^2^	793 (180	497 to 1,213
GEDVIfem-avg, mL/m^2^	1,034 (275	599 to 1,646
EVLWIjug-avg, mL/kg	10.71 (3.43	4 to 18
EVLWIfem-avg, mL/kg	11.54 (3.89	4 to 20
RMSCV CIjug	0.06	
RMSCV CIfem	0.05	
RMSCV GEDVIjug	0.06	
RMSCV GEDVIfem	0.05	
RMSCV EVLWIjug	0.06	
RMSCV EVLWIfem	0.07	
CVPjug-avg, mmHg	16.1 (5.4	4 to 27
CVPfem-avg, mmHg	17.7 (5.7	6 to 34
Sinus rhythm, n	22 (92%)	
Atrial fibrillation, n	2 (8%)	
Mechanical ventilation, n	18 (75%)	
Catecholamine therapy, n	16 (67%)	
Sinus rhythm + controlled ventilation	8 (33%)	

**Reason for ICU admission**		

Pneumonia, acute respiratory insufficiency, n	7 (29%)	
cirrhosis of the liver/liver failure, n	6 (25%)	
gastrointestinal bleeding, n	4 (17%)	
need for cardiopulmonary resuscitation, n	3 (13%)	
sepsis, n	2 (8%)	
pancreatitis, n	2 (8%)	

**Vascular access**		

arterial line right femoral artery, n	13 (54%)	
arterial line left femoral artery, n	11 (46%)	
CVC right jugular vein, n	14 (58%)	
CVC left jugular vein, n	10 (42%)	
CVC right femoral vein, n	16 (67%)	
CVC left femoral vein, n	8 (33%)	
CVC and arterial line on same side	14 (58%)	
CVC and arterial line on different sides	10 (42%)	

SAPS II, Simplified Acute Physiology Score; TISS, Therapeutic Intervention Scoring System; CIjug-avg, average of jugular cardiac index; CIfem-avg, average of femoral cardiac index; GEDVIjug-avg, average of jugular global end-diastolic volume index; GEDVIfem-avg, average of femoral global end-diastolic volume index; EVLWIjug-avg, average of jugular extra-vascular lung water index; EVLWIfem-avg, average of femoral extra-vascular lung water index; RMSCV, root mean square coefficient of variation; CVPjug-avg, average of jugular central venous pressure; CVPfem-avg, average of femoral central venous pressure; CVC, central venous catheter.

### TPTD, vascular access

TPTD variables were calculated from TPTD curves after jugular injection (TPTD variable jug) and femoral injection (TPTD variable fem).

Basic cardiopulmonary characteristics, variability of single TPTD measurements (root mean square coefficient of variation) and data concerning site of vascular access are depicted in Table 1.

### Effect of catheter site on TPTD measurements

**Figure 1:**
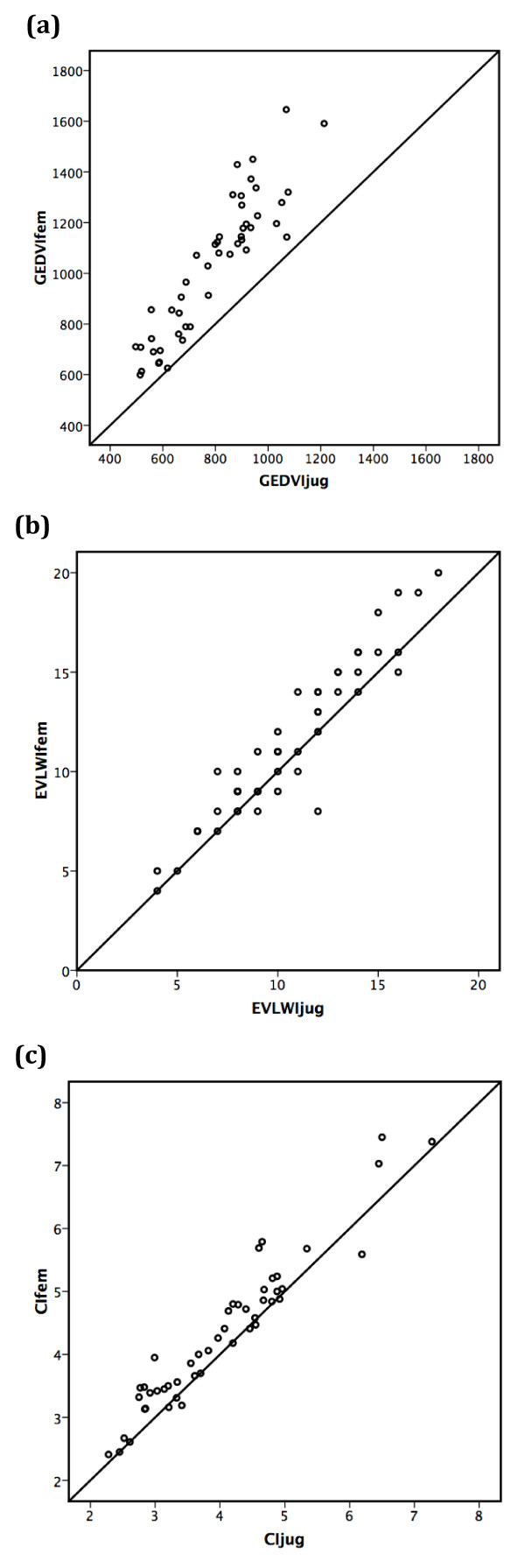
**Correlation of femoral and jugular transpulmonary thermodilution variables**. Scatter plot showing the correlation of femoral and jugular global end-diastolic volume index (r_m _= 0.88; *P *< 0.001) **(a)**, femoral and jugular extra-vascular lung water index (r_m _= 0.93; *P *< 0.001) **(b)**, and femoral and jugular cardiac index (r_m _= 0.95; *P *< 0.001) **(c)**. GEDVIjug, jugular global end-diastolic volume index (mL/m^2^); GEDVIfem, femoral global end-diastolic volume index (mL/m^2^); EVLWIjug, jugular extra-vascular lung water index (mL/kg); EVLWIfem, femoral extra-vascular lung water index (mL/kg); CIjug, jugular cardiac index (L/min/m^2^); CIfem, femoral cardiac index (L/min/m^2^).

**Figure 2:**
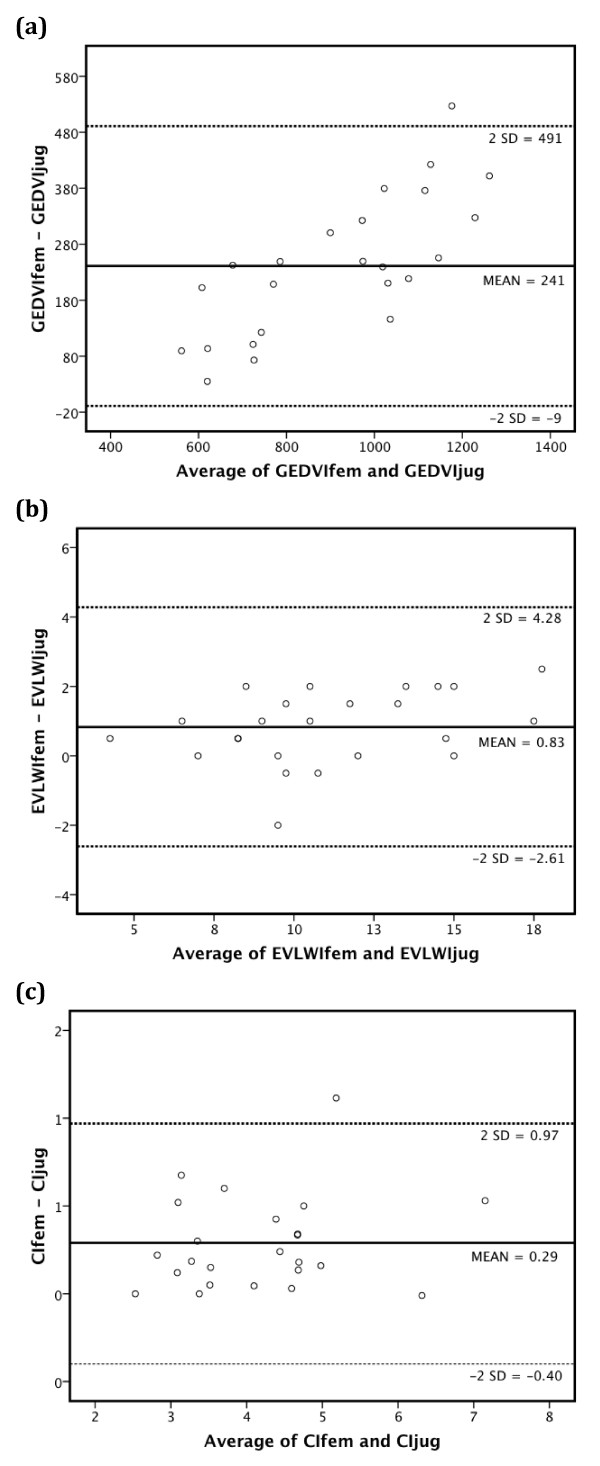
**Transpulmonary thermodilution after femoral and jugular injection: Bland-Altman analysis**. Bland-Altman analysis of global end-diastolic volume index **(a)**, extra-vascular lung water index **(b) **and cardiac index **(c) **derived from transpulmonary thermodilution after femoral and jugular injection. GEDVIjug, jugular global end-diastolic volume index (mL/m^2^); GEDVIfem, femoral global end-diastolic volume index (mL/m^2^); EVLWIjug, jugular extra-vascular lung water index (mL/kg); EVLWIfem, femoral extra-vascular lung water index (mL/kg); CIjug, jugular cardiac index (L/min/m^2^); CIfem, femoral cardiac index (L/min/m^2^). The solid line indicates the mean difference between variables determined after femoral and jugular injection. The dotted lines indicate the limits of agreement (2*SD).

GEDVIfem and GEDVIjug were highly significantly correlated (r_m _= 0.88; b = 1.32 ± 0.11, *P *< 0.001), but their means were significantly different (1,034 ± 275 vs. 793 ± 180 mL/m^2^; *P *< 0.001) (Figure 1a). Bland-Altman analysis resulted in a bias of +241 mL/m^2 ^and limits of agreement of -9 and +491 mL/m^2 ^(Figure 2a, Table 2).

**Table 2:** Bias and 95% limits of agreement of variables derived from femoral and jugular transpulmonary thermodilution

TPTD fem vs. jug	Bias	95% limits of agreement	Percentage error
GEDVIfem vs. GEDVIjug	+241 mL/m^2^	-9 mL/m^2^ +491 mL/m^2^	-
EVLWIfem vs. EVLWIjug	+0.83 mL/kg	-2.61 mL/kg +4.28 mL/kg	-
CIfem vs. CIjug	+0.29 L/min/m^2^	-0.40 L/min/m^2^ +0.97 L/min/m^2^	16%

TPTD, transpulmonary thermodilution; GEDVIfem, femoral global end-diastolic volume index; GEDVIjug, jugular global end-diastolic volume index; EVLWIfem, femoral extra-vascular lung water index; EVLWIjug, jugular extra-vascular lung water index; CIfem, femoral cardiac index; CIjug, jugular cardiac index.

**Figure 3:**
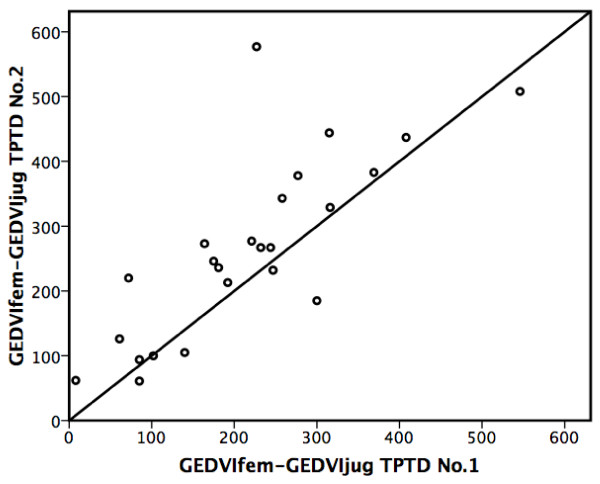
**Intra-individual correlation of the two pairs of transpulmonary thermodilution measurements**. Scatter plot demonstrating significant intra-individual correlation of the two pairs of transpulmonary thermodilution measurements (No. 1 and No. 2) in each patient (r = 0.79; *P *< 0.001). GEDVIjug, jugular global end-diastolic volume index (mL/m^2^); GEDVIfem, femoral global end-diastolic volume index (mL/m^2^); GEDVIfem - GEDVIjug, difference between GEDVI values after femoral and jugular injection; TPTD, transpulmonary thermodilution.

Comparison of the two pairs of measurements in each patient demonstrated a significant intra-individual correlation of the differences (GEDVIfem-GEDVIjug) (r = 0.79; *P *< 0.001) (Figure 3).

We performed GEE-regression analyses to characterize the main factors significantly associated with the difference (GEDVIfem-GEDVIjug).

**Figure 4:**
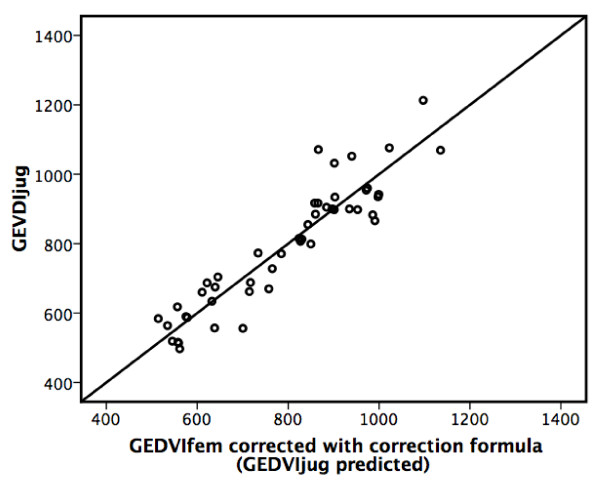
**Femoral global end-diastolic volume index corrected by the correction formula**. Scatter plot illustrating the predictive capability of the correction formula of jugular global end-diastolic volume index (adjusted r^2 ^= 0.75). GEDVIjug, jugular global end-diastolic volume index (mL/m^2^).

Bivariate correlation analyses suggested an association of the difference (GEDVIfem-GEDVIjug) with height (r_m _= 0.32; b = 4.8 ± 2.2, *P *= 0.031), normal body weight (BW) (r_m _= 0.32; b = 4.8 ± 2.2, *P *= 0.031), GEDVIfem (r_m _= 0.87; b = 0.42 ± 0.05, *P *< 0.001) and GEDVIjug (r_m _= 0.58; b = 0.32 ± 0.11, *P *= 0.005). Furthermore, co-linearity of height and BW was demonstrated with ideal BW (IBW) as the parameter with the strongest association to the difference (GEDVIfem-GEDVIjug). Therefore, GEDVIfem, CIfem and IBW were included in generalized linear models to characterize factors independently associated with the difference (GEDVIfem-GEDVIjug). The final model including GEDVIfem (*P *< 0.001), CIfem (*P *= 0.011) and IBW (*P *= 0.162) resulted in the prediction formula of GEDVIjug with the highest predictive capability (adjusted r^2 ^= 0.75) (Figure 4):

(GEDVIjug, jugular global end-diastolic volume index (mL/m^2^); GEDVIfem, femoral global end-diastolic volume index (mL/m^2^); CIfem, femoral cardiac index (L/min/m^2^); IBW, ideal body weight (kg)).

We calculated sensitivity, specificity, positive predictive value (PPV), negative predictive value (NPV) and accuracy for prediction of elevated GEDVIjug (>800 mL/m^2^) and decreased GEDVIjug (<680 mL/m^2^) based on uncorrected GEDVIfem, GEDVIfem corrected by subtraction of the mean bias of +241 mL/m^2 ^as well as GEDVIfem corrected by the correction formula (Table 3). Although even uncorrected GEDVIfem resulted in acceptable predictive capabilities, correction resulted in further improvement of the prediction of GEDVIjug.

**Table 3:** Predictive capabilities of uncorrected and corrected femoral global end-diastolic volume index

	Uncorrected GEDVIfem	GEDVIfem - mean bias (GEDVIfem - GEDVIjug)	GEDVIfem corrected by the correction formula
Diagnostic accuracy	67%	90%	88%
Sensitivity elevated GEDVIjug	100	100	100
Specificity elevated GEDVIjug	61	91	96
PPV elevated GEDVIjug	74	93	96
NPV elevated GEDVIjug	100	100	100
Sensitivity decreased GEDVIjug	31	100	81
Specificity decreased GEDVIjug	100	91	94
PPV decreased GEDVIjug	100	84	87
NPV decreased GEDVIjug	74	100	91

Diagnostic accuracy, sensitivity, specificity, positive predictive value, negative predictive value for the prediction of jugular global end-diastolic volume based on uncorrected femoral global end-diastolic volume index, femoral global end-diastolic volume index corrected by subtraction of the mean bias and femoral global end-diastolic volume index corrected by the correction formula.

GEDVIfem, femoral global end-diastolic volume index; GEDVIjug, jugular global end-diastolic volume index; PPV, positive predictive value; NPV, negative predictive value. Elevated GEDVI means GEDVI >800 mL/m^2 ^and decreased GEDVI means GEDVI <680 mL/m^2^

To evaluate the usefulness of the correction formula derived from the first 24 patients following the study period we studied five more consecutive patients with superior and inferior vena cava access at the same time as a control population (four males, one female; mean age 57.2 ± 9.0 years, mean height 178 ± 13 cm, mean weight 93.6 ± 20.2 kg; two patients died on ICU, three patients survived ICU stay, reason for ICU admission: pancreatitis in two patients, cirrhosis of the liver in two patients, pneumonia in one patient). Mean GEDVIfem and GEDVIjug in these patients was 896 ± 126 mL/m^2 ^and 720 ± 76 mL/m^2^, respectively. The mean difference between GEDVIfem and GEDVIjug (bias) in this control group was 20% of GEDVIfem (176 mL/m^2^). In this group correction of GEDVIfem by subtraction of the mean bias of +241 mL/m^2 ^(mean bias in the study group) resulted in a reduction of the mean difference to 7% (65 mL/m^2^). A further reduction of the bias to 6% (50 mL/m^2^) was achieved using the correction formula. Uncorrected GEDVIfem had a diagnostic accuracy for prediction of elevated GEDVIjug (>800 mL/m^2^) and decreased GEDVIjug (<680 mL/m^2^) of only 20%. Correction of GEDVIfem by subtraction of the mean bias of +241 mL/m^2 ^resulted in an accuracy of 60%. However, a diagnostic accuracy of 70% in this control population could be achieved when GEDVIfem was corrected by the correction formula.

The comparison of EVLWIfem and EVLWIjug demonstrated that EVLWIfem and EVLWIjug were highly significantly correlated (r_m _= 0.93; b = 1.07 ± 0.05, *P *< 0.001), but significantly different (11.54 ± 3.89 vs. 10.71 ± 3.43 mL/kg; *P *< 0.001) (Figure 1b).

In Figure 2b and Table 2 Bland-Altman analysis for the comparison of EVLWIfem and EVLWIjug is depicted (bias +0.83 mL/kg; limits of agreement -2.61 and +4.28 mL/kg).

Bivariate correlation analyses suggested an association of the difference (EVLWIfem-EVLWIjug) with EVLWIfem (r_m _= 0.50; b = 0.19 ± 0.04, *P *< 0.001) and CIfem (r_m _= -0.46; b = -0.25 ± 0.10, *P *= 0.015).

Regarding a co-linearity of height and adjusted BW, the final generalized model included adjusted BW, EVLWIfem and CIfem to characterize factors independently associated with the difference (EVLWIfem-EVLWIjug).

Including the independently predictive factors EVLWIfem (*P *< 0.001) and CIfem (*P *= 0.014) that were associated with the difference (EVLWIfem-EVLWIjug) resulted in a prediction formula of EVLWIjug (adjusted r^2 ^= 0.34):

(EVLWIjug, jugular extra-vascular lung water index (mL/kg); EVLWIfem, femoral extra-vascular lung water index (mL/kg); CIfem, femoral cardiac index (L/min/m^2^)).

CI was calculated after femoral injection (CIfem) and jugular injection (CIjug). Figure 1c shows that CIfem and CIjug were significantly different (4.31 ± 1.18 vs. 4.03 ± 1.13 L/min/m^2^; *P *< 0.001) but highly significantly correlated (r_m _= 0.95; b = 0.99 ± 0.04, *P *< 0.001).

Bland-Altman analysis revealed a bias of +0.29 L/min/m^2 ^with lower/upper limit of agreement of -0.40 and +0.97 L/min/m^2 ^(Figure 2c, Table 2). The percentage error was 16%. The final prediction model for CIjug based on GEDVIfem (*P *< 0.001) and CVPfem (*P *= 0.004) demonstrated a substantial fit (adjusted r^2 ^= 0.49) with the correction formula:

(CIjug, jugular cardiac index (L/min/m^2^); CIfem, femoral cardiac index (L/min/m^2^); GEDVIfem, femoral global end-diastolic volume index (mL/m^2^); CVPfem, femoral central venous pressure (mmHg); height (cm)).

## Discussion

Regarding the importance of GEDVI, EVLWI and CI we investigated the accuracy of TPTD measurements using femoral injection of the TPTD bolus instead of the gold standard injection sites via superior vena cava access. We found a highly significant correlation of GEDVI, EVLWI and CI determined after femoral injection compared to simultaneous measurements via jugular access. The bias for EVLWI and CI was low (with a low percentage error for CI). Uncorrected EVLWIfem and CIfem had high predictive capabilities for the normal ranges as well as for pathological values of EVLWIjug and CIjug. Using correction formulas derived from our data further improved the predictive capabilities.

Regarding GEDVI, a significant and self-explaining bias was expected according to the principle of GEDVI determination. GEDVI is calculated as 0.8*(ITTV - EVLWI) with ITTV (intrathoracic thermal volume) being the total volume participating in indicator dilution between the tip of the venous injection site and the tip of the arterial TPTD detection site. Injection of the indicator in the distal inferior vena cava adds the volume of the inferior vena cava to the total volume participating in thermodilution, resulting in an artificial increase in mean transit time and ITTV.

Therefore it was a further aim of our study to develop a correction formula of GEDVIjug compensating GEDVIfem for the bias (GEDVIfem-GEDVIjug) and factors independently associated with the bias.

Simple subtraction of the mean bias of +241 mL/m^2 ^from GEDVIfem resulted in high sensitivity, specificity, PPV, NPV and accuracy regarding decreased as well as increased GEDVIjug. Correction of GEDVIfem using the correction formula resulted in even higher predictive capabilities, emphasizing a certain robustness of the formula in the study population as well as in the group of the five more consecutive patients studied as a control population.

Interestingly, the mean difference of GEDVIfem and GEDVIjug was about 100 mL higher than in the study of Schmidt et al. [24]. However, the number of patients in this study was not high and there were multiple measurements (one to nine per patients) included in the results. Therefore, it can not be excluded that the bias in this study was influenced by multiple measurements in a patient with a smaller difference of (GEDVIfem-GEDVIjug). Regarding the additional volume of parts of the inferior vena cava participating in TPTD, this also could be related to the different height of the patient population as well as to the different preload conditions. Despite no access to the original data of Schmidt et al., calculation of mean GEDVIfem, mean CIfem and extrapolation of the ideal body weight (based on mean height and three female and eight male patients included in this study) and using these mean data in our formula would have estimated GEDVIjug 792.65 mL instead of 876.85 mL with a mean bias of 84.2 mL. This is a reduction of 56.5 mL or 40% compared to the bias of 140.73 mL for uncorrected GEDVIfem, thus suggesting a certain usefulness of the formula in different patient populations.

Regarding EVLWI we found even better bias, accuracy and other predictive capabilities of EVLWIfem with respect to EVLWIjug. Regarding theoretical considerations with EVLWI based on the downslope time of the thermodilution curve this finding is not surprising. The downslope time, a linear part of the thermodilution curve, is determined by the largest compartment of the different series-connected compartments participating in the dilution of the TPTD indicator bolus. Since the volume of this compartment (pulmonary thermovolume, PTV) comprising EVLW and pulmonary blood volume (PBV) theoretically is not influenced by the addition of a further compartment (inferior vena cava) between the injection site (inferior vena cava) and the right atrium, the bias should be close to zero. Considering the calculation of EVLW based on subtraction of PBV from PTV estimating PBV 25% of GEDV, a small systematic bias of uncertain clinical relevance could be postulated. However, despite a small but significant difference of EVLWIjug and EVLWIfem, considering high predictive capabilities of EVLWIjug using EVLWIfem, this small difference seems to be without clinical relevance.

Similar considerations apply for the comparison of CIfem and CIjug. Uncorrected CIfem showed high predictive capabilities for CIjug. A small bias of 0.29 L/min/m^2 ^and a percentage error as low as 16% show that uncorrected CIfem can be used for the assessment of cardiac output in the setting of critically ill ICU patients. The small bias and the low percentage error are in line with theoretical considerations that the area under the curve of the thermodilution curve determining CI should not substantially be affected by injection of the indicator in the femoral vein.

These findings seem to be of importance in daily clinical practice since CVC insertion via superior vena cava access is not feasible in several critically ill patients who need to be monitored using advanced hemodynamic monitoring: Thrombosis of jugular or subclavian veins or use of these veins for dialysis catheters can make it impossible to use superior vena cava access for CVC placement. Furthermore, for emergency central venous access and in burn patients as well as patients with contraindication for Trendelenburg position (neurologic/neurosurgery patients, heart insufficiency), CVC insertion via the femoral vein can be of special importance [31,32].

### Limitations of the study

Despite a higher number of patients included and providing a constant number of measurements in each patient compared to previous data, our study was performed in a limited number of patients in the study population. The study was performed monocentric in a medical ICU. Moreover, the number of patients in the control population is small. Furthermore, our study population contained only one patient with severe obesity (BMI >35 kg/m^2^) and one patient with underweight (BMI <18.5 kg/m^2^).

Despite encouraging application to our control collective and to previous data, the correction formulas in particular have to be confirmed in future investigations of different patient populations and in multicentric studies.

## Conclusions

TPTD after injection of the thermo-bolus through a femoral CVC provides precise data on GEDVI with a high correlation but a self-evident significant bias related to the augmented TPTD-volume. After correction of GEDVIfem using a correction formula, GEDVIfem shows high predictive capabilities for GEDVIjug. Regarding CI and EVLWI accurate TPTD-data is obtained using femoral access.

These data seem to be of importance regarding an underestimated frequency of femoral central venous access, particularly in emergency situations, malfunction of variability parameters (such as stroke volume variation (SVV)) in numerous patients requiring hemodynamic monitoring devoid of sinus rhythm and controlled ventilation, and numerous studies emphasizing the clinical usefulness of volumetric parameters such as GEDVI and EVLWI.

## Key messages

• TPTD after injection of the indicator bolus via a femoral central venous catheter provides precise data on GEDVI with a high correlation but significant bias related to the augmented thermodilution volume.

• A correction formula for jugular GEDVI after femoral TPTD-indicator injection was calculated.

• After correction of GEDVIfem using the correction formula, GEDVIfem shows high predictive capabilities for GEDVIjug.

• For determination of CI and EVLWI accurate TPTD-data is obtained using femoral access for indicator injection.

## Abbreviations

BW: body weight; CI: cardiac index; CIfem: cardiac index after femoral injection of the indicator bolus; CIjug: cardiac index after jugular injection of the indicator bolus; CVC: central venous catheter; CVP: central venous pressure; EVLW: extra-vascular lung water; EVLWI: extra-vascular lung water index; EVLWIfem: extra-vascular lung water index after femoral injection of the indicator bolus; EVLWIjug: extra-vascular lung water index after jugular injection of the indicator bolus; GEDV: global end-diastolic volume; GEDVI: global end-diastolic volume index; GEDVIfem: global end-diastolic volume index after femoral injection of the indicator bolus; GEDVIjug: global end-diastolic volume index after jugular injection of the indicator bolus; GEE: general estimation equation; IBW: ideal body weight; ICU: intensive care unit; ITTV: intrathoracic thermal volume; NPV: negative predictive value; PBV: pulmonary blood volume; PPV: positive predictive value; PTV: pulmonary thermovolume; r_m_: Spearman correlation coefficient; RMSCV: root mean square coefficient of variation; SAPS II: Simplified Acute Physiology Score II; SD: standard deviation; SE: standard error; SVV: stroke volume variation; TPTD: transpulmonary thermodilution; TDTDfem: transpulmonary thermodilution variable after femoral injection of the indicator bolus; TDTDjug: transpulmonary thermodilution variable after jugular injection of the indicator bolus; TISS: Therapeutic Intervention Scoring System.

## Competing interests

The authors declare that they have no competing interests.

## Authors' contributions

BS, AU and VP contributed to the conception and design of the study. They were responsible for acquisition, analysis and interpretation of data. BS drafted the manuscript. RMS and WH participated in study design and coordination and helped to draft the manuscript. TS participated in the design of the study and performed the statistical analysis. All authors read and approved the final manuscript.
